# Perception of faculty in the community health sciences colleges towards simulation-based education in clinical nutrition undergraduate practical courses

**DOI:** 10.1186/s12909-024-05338-w

**Published:** 2024-04-11

**Authors:** Iman Abdullah Bindayel, Shahad Ahmed Alahmad

**Affiliations:** Department of Community Health Sciences, College of Applied Medical Sciences, King Saud University, Riyadh, Saudi Arabia

**Keywords:** Simulation, Simulation-based education, Undergraduate, Nutrition, Dietetics, Faculty, Faculty members

## Abstract

**Background:**

Simulation now is widely used for training and education in different fields including healthcare education. Medicine and healthcare students can be trained in a secure, efficient, and engaging setting by Simulation-based Education (SBE). Therefore, this study aimed to assess the perception of faculty members in the community health departments towards SBE to be used in practical subjects for clinical nutrition undergraduate courses.

**Method:**

This cross-sectional survey was conducted among community health sciences faculty members. The perception was assessed using a self-administered questionnaire that included three sections.

**Results:**

This questionnaire was completed by 125 faculty members, of whom 36 (28.8%) were male and 89 (71.2%) were female. Overall, faculty members had positive perceptions, with a mean score of 3.86 ± 0.74, but a high level of anxiety toward SBE, with a mean score of 3.42 ± 0.75. There was a statistically significant difference between the responses of the faculty members based on the training they received in simulation (*P* = 0.001).

**Conclusion:**

The study results indicate that community health sciences faculty members’ perception of SBE in Saudi Arabia is generally positive. However, the results show high levels of anxiety among faculty members toward SBE.

## Background

Medicine and healthcare students can be trained in a secure, efficient, and engaging setting thanks to Simulation-based Education (SBE) [1]. A training or educational technique known as SBE uses guided experiences to supplement or replace real-world experience [2]. It is defined as an educational strategy based on learning theories rather than technology [1]. Simulation aims to interactively recreate elements of the real world so that learners can fully immerse themselves in the learning environment [2, 3]. Simulators have long been used in aviation and the military to train employees in technical skills and safety-related attitudes (such as teamwork and communication) [4]. There are various simulation techniques available [5]. The most prevalent techniques are role-playing, standardized or simulated patients, computerized mannequins, and virtual simulations [5]. SBE has been the subject of extensive study and research for over two decades [5]. A significant portion of the undergraduate curriculum now includes simulation in the healthcare sciences as an evidence-based, efficient learning tool [5].

Simulation in medical healthcare practice and education has been applied since the early twentieth century [6, 7]. Unlike simulation in medicine, nursing, and other healthcare sectors, simulation in nutrition and dietetics began to see the light in the late 70s [8]. Most of the research was conducted in the United States. In 1979, The University of Connecticut used simulation exercises for interview training in dietetics to develop counseling and interviewing skills for the students [8]. In the same year, Syracuse University applied a course in the first semester of the junior year, which involved a simulated team conference for nursing and dietetics students to educate them about the healthcare team [9]. In 1981, at The University of Pennsylvania, the students watched videotaped simulated interviews to learn counseling and communication skills in the nutrition course [10]. In the mid-80s, The University of Pittsburgh used simulation interviews to evaluate students` clinical skills for nutrition counseling [11]. This evaluation aimed to assess graduate students` clinical skills to resolve issues with dietary adherence [11]. During the same period, The University of Kentucky developed a learning technique for senior dietetics students to prepare them for the future [12]. This learning activity included scenarios and simulation games where the students selected a career choice and discussed the options created by the scenarios [12]. In the late 80s, The University of Nebraska provided a nutrition workshop for 30 managers of government-sponsored nutrition centers for the elderly in different areas of the country [13]. The workshop involved videotaped simulations of typical nutritional issues experienced by older adults in these centers [13]. As an outcome, the knowledge increased, and 75% of the managers benefited and applied the ideas from the workshop [13]. In the 1990s, the research surrounding simulation in nutrition and dietetics began to appear and continued to increase until today [14]. Furthermore, the interest and demand for simulation in medical education have increased due to its advantages, including providing safe and effective patient care, reducing medical errors, experiential learning, deliberate practice, transformative learning, and debriefing techniques [15]. Despite that, simulation application in nutrition and dietetics is underexplored compared with other medical fields where simulation has been practiced for decades [14].

A systematic review published in 2019 and included fourteen studies about simulation in dietetics demonstrated that using simulated patients was essential for building and developing counseling skills and readiness for practice [16]. Moreover, simulated patients could be used to assess counseling skills in Objective Structured Clinical Examination (OSCE) exams [16]. Since the decision-making in dietetics depends on evidence-based practice, the development and application of evaluation methods are needed to support the evidence base for simulation skill acquisition in dietetics education [16]. Eventually, further research about simulation in dietetics and nutrition is needed to determine the skills likely to be developed and enhanced by simulation [16].

There are only a few studies in the last five years that were published about the use of simulation in nutrition and dietetics training or education worldwide [17–25]. As is the case worldwide, to the best of our knowledge, only a few studies were done in Saudi Arabia about the perception towards simulation in education, either among faculty members or students in health colleges [1, 26]. Consequently, as far as we know, this is the first study in Saudi Arabia that assessed faculty perception towards SBE in the community health sciences departments. It is suggested that the use of simulation in education will improve students` self-esteem, skills, critical thinking, teamwork, essential procedural skills, and decision-making, likewise increasing patients` safety and reducing medical errors [15]. Based on the MedTeams study, simulation practice decreased medical errors by 26.5% [27]. Moreover, most studies about simulation and nutrition published in the last ten years were conducted among students, athletes, and the general population. Thus, this study aimed to assess faculty members` perception in the community health departments towards SBE to be used in practical subjects for clinical nutrition undergraduate courses, to identify faculty members’ perceived barriers toward the integration of SBE, and to fill the gap regarding SBE in nutrition.

## Methods

### Study setting

This is a cross-sectional survey in which Saudi universities that provided a Clinical Nutrition program in Saudi Arabia were targeted during the last semester of the academic year 2022–2023. The study surveyed faculty members from the Colleges of Applied Medical Sciences (CAMS) in 16 universities. Universities that are included in this study are King Saud University, King Saud bin Abdulaziz University for Health Sciences, Princess Nourah bint Abdulrahman University, Umm Al-Qura University, King Abdulaziz University, King Faisal University, Qassim University, Taibah University, University of Hail, Jazan University, Al Baha University, Northern Borders University, Imam Abdulrahman Bin Faisal University, University of Hafr al Batin, University of Tabuk, and Shaqra University.

Faculty were invited via university email to fill out a self-administered questionnaire. Social media platforms such as LinkedIn, WhatsApp, and Twitter were used to distribute the questionnaire among faculty using a snowball sampling technique. The study will identify the universities by pseudonym, as well as, the anonymity of the participants to ensure confidentiality.

### Subjects

In this cross-sectional survey, faculty members from different universities in Saudi Arabia were approached. Faculty members from both genders who have taught clinical nutrition practical subjects and who have had at least two years of experience in their academic work were included. Faculty members who had never taught clinical nutrition practical subjects were excluded. Furthermore, the exclusion criteria included newly employed faculty members.

G*Power 3 [28] was used to determine the sample size that will allow for the assessment of meaningful associations and the detection of effect sizes (small, medium, or large). Using the one-way ANOVA test and an alpha value of 0.05, the results indicated that with a power of 0.95, sample sizes of 1865, 305, and 125 were needed to detect effect sizes of 0.10 (small), 0.25 (medium), and 0.40 (large), respectively. Reaching the study’s sample size of 125 meant that large effect sizes could be detected in the statistical analyses.

### Data collection

The questionnaire used for this study was developed by Ahmed et al. [26], which was inspired by literature [2, 28–30] and resulted from discussions with medical teachers. The questionnaire was subjected to a pilot sample of 35 faculty members before being distributed in its final form. The data was initially treated to verify the psychometric characteristics of the questionnaire, so the reliability coefficient of Cronbach’s alpha was extracted for the questionnaire dimensions separately. Then, the overall reliability of the questionnaire was calculated. The reliability coefficient of Cronbach’s alpha showed that the questionnaire has good reliability coefficients ranging from the value (0.86) to (0.90), that have located in the range of excellent reliability coefficients (0.80–1) identified by [32], which makes it valid to achieve the objectives of the study. In general, the result showed that the reliability of the overall questionnaire is (0.88), which means that it is possible to obtain identical results by (88%) between this application and re-application of this questionnaire, and this implicitly means that the items are clear and explicit and carry accurate ideas for which the respondent’s understanding of it does not vary with time. Pearson correlation coefficients were calculated to examine the correlation of the questionnaire’s items with the dimensions to which they belong to ensure the structural validity of the questionnaire. The result showed that the correlation coefficients of the items with their affiliated dimensions are significant correlations at the level of significance (0.01), which indicates a high internal validity of the dimensions of the questionnaire. The items associated with the total average of the responses of the dimensions are considered valid expressions that measure what they were set for.

The questionnaire, in its final form, consisted of [27] items, which were divided into four main dimensions: 1st faculty members’ perceptions toward simulation-based education, 2nd determine anxiety of faculty members toward simulation-based education, 3rd faculty members’ perception toward the integration of simulation in education, and 4th faculty members’ perceived barriers to simulation-based education in undergraduate curricula.

At the beginning of the questionnaire form, a brief paragraph about the study and the study’s aim helped introduce the respondents to the study. To obtain consent, the respondents agreed to participate by clicking a button for participation agreement before answering the questionnaire. The survey was distributed in English, and it involved three parts. The first part included socio-demographic data of respondents such as gender, age, education, occupation, level of experience, teaching university, formal training for SBE, and exposure to SBE during teachers’ clinical nutrition education. The second part included some questions to assess teachers’ perceptions of SBE and integrate it into their teaching. Answers from the respondents were read on a five-point Likert-type scale. In the last part, an open-ended question was asked to explore faculty members’ perceived barriers toward SBE. Faculty were invited via university email to fill out a self-administered questionnaire. Social media platforms such as LinkedIn, WhatsApp, and Twitter were used to distribute the questionnaire among faculty using a snowball sampling technique. The study will identify the universities by pseudonym, as well as the anonymity of the participants to ensure confidentiality.

### Statistical analysis

After the data collection, data were organized, checked, and analyzed with the Statistical Package for the Social Sciences (SPSS) version 25. Frequencies and percentages expressed the number of participants in the socio-demographic variables. Descriptive statistics, t-test, one-way ANOVA, chi-square test, means, and standard deviation were used for data analysis. A P-value < 0.05 will be considered to be statistically significant.

### Likert’s scale intervals

The length of the intervals in the five-point Likert scale was determined by calculating the range (5 − 1 = 4) and then dividing it by the most significant value in the scale to get the length of the interval (4 ÷ 5 = 0.8), then this value was added to the lowest value in the scale (the beginning of the scale, which is an integer one), to determine the upper limit of this interval. Table 1. shows Likert’s scale intervals.

**Table 1:** Likert’s scale intervals

Verbal scale	Strongly Agree	Agree	Neutral	Disagree	Strongly disagree
**Perception degree**	Very positive	Positive	Moderate	Negative	Very negative
**Anxiety degree**	Very high	High	Moderate	Low	Very low
**Numeric scale**	5	4	3	2	1
**Scale intervals**	More than 4.20	From 3.40 to less than 4.20	From 2.60 to less than 3.40	From 1.80 to less than 2.60	Less than 1.80

## Results

### Socio-demographic data of the sample

A total of 125 faculty members filled out the questionnaire. Table 2. shows the distribution of the participants according to the study variables. The majority were females (71.2%), and (93.6%) aged less than 60 years. Most participants held either a Master’s degree (45.6%) or followed by Doctorate (43.2%) and none of the respondents had postgraduate diplomas. Most of the participants had more than five years of experience. 68% of participants did not receive formal simulation training (*P* = 0.001), and 62.4% were not exposed to SBE during their education.

**Table 2:** Study sample socio-demographic data (*N* = 125)

Variable	N (%)	Total N (%)	Mean score (SD)	*p*-Value
**Gender**				
Male	36 (28.8%) 89 (71.2%)	125 (100%)	3.54 (0.57) 3.67 (0.55)	0.244
Female				
**Age**				
20–39	62 (49.6%) 55 (44%) 8 (6.4%)	125 (100%)	3.53 (0.57) 3.73 (0.56) 3.72 (0.29)	0.117
40–59				
+ 60				
**Education**				
Postgraduate certificates	14 (11.2%) - 57 (45.6%) 54 (43.2%)	125 (100%)	3.65 (0.54) - 3.65 (0.50) 3.61 (0.62)	0.916
Postgraduate diplomas				
Master’s degrees				
Doctorates				
**Occupation**				
Teaching Assistant	15 (12%) 32 (25.6%) 38 (30.4%) 25 (20%) 15 (12%)	125 (100%)	3.55 (0.45) 3.60 (0.60) 3.67 (0.62) 3.72 (0.43) 3.54 (0.60)	0.822
Lecturer				
Assistant Professor				
Associate Professor				
Professor				
**Level of experience**				
Less than 5 years	51 (40.8%) 41 (32.8%) 33 (26.4%)	125 (100%)	3.65 (0.57) 3.60 (0.50) 3.64 (0.61)	0.915
6–10 years				
More than 11 years				
**Teaching university**				
A	6 (4.8%) 6 (4.8%) 14 (11.2%) 9 (7.2%) 7 (5.6%) 9 (7.2%) 15 (12%) 5 (4%) 9 (7.2%) 5 (4%) 6 (4.8%) 8 (6.4%) 6 (4.8%) 9 (7.2%) 4 (3.2%) 7 (5.6%)	125 (100%)	3.43 (0.48) 3.74 (0.89) 3.64 (0.56) 3.81 (0.60) 3.63 (0.59) 3.68 (0.67) 3.56 (0.55) 3.38 (0.69) 3.68 (0.65) 3.44 (0.58) 3.93 (0.31) 3.62 (0.30) 3.71 (0.55) 3.46 (0.65) 3.53 (0.42) 3.78 (0.39)	0.957
B				
C				
D				
E				
F				
G				
H				
I				
J				
K				
L				
M				
N				
O				
P				
**Received training for simulation**				
Yes	40 (32%) 85 (68%)	125 (100%)	3.89 (0.45) 3.51 (0.56)	0.001*
No				
**Exposed to SBE during education**				
Yes	47 (37.6%) 78 (62.4%)	125 (100%)	3.71 (0.55) 3.58 (0.56)	0.201
No				

* Significant difference at the level (0.05) or less for t-test and one-way ANOVA. (SD): Standard Deviation

**Table 3:** Received training for simulation and socio-demographic data

Variable	Received training for simulation		*p*-Value
	Yes N (%)	No N (%)	
**Gender**			0.099
Male	8 (20.0%)	28 (32.9%)	
Female	32 (80.0%)	57 (67.1%)	
**Age**			*0.016
20–39	13 (32.5%)	49 (57.6%)	
40–59	25 (62.5%)	30 (35.3%)	
+ 60	2 (5%)	6 (7.1%)	
**Education**			*0.012
Postgraduate certificates	3 (7.5%)	11 (12.9%)	
Master’s degrees	26 (65%)	31 (36.5%)	
Doctorates	11 (27.5%)	43 (50.6%)	
**Occupation**			*0.015
Teaching Assistant	3 (7.5%)	12 (14.1%)	
Lecturer	9 (22.5%)	23 (27.1%)	
Assistant Professor	8 (20%)	30 (35.3%)	
Associate Professor	10 (25%)	15 (17.6%)	
Professor	10 (25%)	5 (5.9%)	
**Years of experience**			0.659
Less than 5 years	17 (42.5%)	34 (40%)	
6–10 years	11 (27.5%)	30 (35.3%)	
More than 11 years	12 (30%)	21 (24.7%)	

*Note* The qualitative variables were compared using the chi-square cross-tabulation test and presented as n (percentage). *A *P* value < 0.05 is considered significant

As shown in Table 3. there was a statistically significant difference in age between received training for simulation or not (*P* = 0.016); 62.5% of those who received formal training for simulation were aged 40–59 years, whereas 57.6% of those who did not were 20–39 years. As well as for training for simulation and educational level (*P* = 0.012), 50.6% of those who did not receive formal training for simulation were with a Doctorate, and 65% of those who received formal training for simulation were with a Master’s degree. Furthermore, a statistically significant difference between received training for simulation and occupation was observed (*P* = 0.015). Associate Professors and Professors were the occupations that received the most formal training for simulation (50%).

**Table 4:** Exposure to SBE during education and socio-demographic data

Variable	Exposure to simulation		*p*-Value
	Yes N (%)	No N (%)	
**Gender**			0.204
Male	11 (23.4%)	25 (32.1%)	
Female	36 (76.6%)	53 (67.9%)	
**Age**			0.210
20–39	22 (46.8%)	40 (51.3%)	
40–59	24 (51.1%)	31 (39.7%)	
+ 60	1 (2.1%)	7 (9%)	
**Education**			*< 0.001
Postgraduate certificates	2 (4.3%)	12 (15.4%)	
Master’s degrees	32 (68.1%)	25 (32.1%)	
Doctorates	13 (27.7%)	41 (52.6%)	
**Occupation**			0.078
Teaching Assistant	4 (8.5%)	11 (14.1%)	
Lecturer	13 (27.7%)	19 (24.4%)	
Assistant Professor	10 (21.3%)	28 (35.9%)	
Associate Professor	10 (21.3%)	15 (19.2%)	
Professor	10 (21.3%)	5 (6.4%)	
**Years of experience**			0.659
Less than 5 years	23 (48.9%)	28 (35.9%)	
6–10 years	12 (25.5%)	29 (37.2%)	
More than 11 years	12 (25.5%)	21 (26.9%)	

*Note* The qualitative variables were compared using the chi-square cross-tabulation test and presented as n (percentage). *A *P* value < 0.05 is considered significant

As shown in Table 4. there was a statistically significant difference between the exposure to simulation during education and educational level (P = < 0.001), ); 68.1% of participants with a Master’s degree were exposed to simulation during schooling. No differences were observed between the exposure to simulation during education and other socio-demographic variables.

### Faculty members’ perceptions toward simulation-based education

Table 5 demonstrates faculty members` perceptions toward SBE in percentages and numbers, which will be shown in detail in the following sections with means and standard deviation.

**Table 5:** Faculty members’ perceptions toward simulation-based education

Order	Questions on three dimensions regarding SBE	Strongly agree N (%)	Agree N (%)	Neutral N (%)	Disagree N (%)	Strongly disagree N (%)	Total N (%)
**Faculty members’ perceptions toward simulation-based education:**							
1	I enjoy my teaching more when I use simulation	27 (21.6%)	57 (45.6%)	32 (25.6%)	2 (1.6%)	7 (5.6%)	125 (100%)
2	Students show more interest when I use simulation tools	35 (28%)	57 (45.6%)	24 (19.2%)	-	9 (7.2%)	125 (100%)
3	Simulation is an effective assessment tool to evaluate students’ learning	37 (29.6%)	56 (44.8%)	23 (18.4%)	-	9 (7.2%)	125 (100%)
4	Simulation-based teaching can improve learning outcomes	49 (39.2%)	49 (39.2%)	14 (11.2%)	-	13 (10.4%)	125 (100%)
5	There is more freedom to learn in a simulated environment than learning in real wards or clinics	24 (19.2%)	48 (38.4%)	35 (28%)	2 (1.6%)	16 (12.8%)	125 (100%)
6	Standardized patient is the best tool for teaching communication skills	24 (19.2%)	62 (49.6%)	23 (18.4%)	-	16 (12.8%)	125 (100%)
7	Simulation-based education improves patient safety	30 (24%)	59 (47.2%)	24 (19.2%)	-	12 (9.6%)	125 (100%)
**Determine anxiety of faculty members toward simulation-based education**:							
8	I need extra support to function effectively in simulation-based teaching	28 (22.4%)	60 (48%)	26 (20.8%)	-	11 (8.8%)	125 (100%)
9	I face problems in managing students in simulated teaching	9 (7.2%)	28 (22.4%)	66 (52.8%)	1 (0.8%)	21 (16.8%)	125 (100%)
10	It takes more time to plan teaching with simulation tools rather than with real patients in student learning	17 (13.6%)	52 (41.6%)	36 (28.8%)	1 (0.8%)	19 (15.2%)	125 (100%)
11	I avoid the integration of simulation in my courses	14 (11.2%)	20 (16%)	49 (39.2%)	7 (5.6%)	35 (28%)	125 (100%)
12	Interaction with standardized patients makes students communicate in an artificial manner with real patients	18 (14.4%)	56 (44.8%)	33 (26.4%)	2 (1.6%)	16 (12.8%)	125 (100%)
**Faculty members’ perception toward the integration of simulation in education**:							
13	Simulation should be a part of the medical curriculum and not a stand-alone activity	29 (23.2%)	61 (48.8%)	16 (12.8%)	3 (2.4%)	16 (12.8%)	125 (100%)
14	Simulation-based activities should be introduced in the undergraduate curriculum from year 1	24 (19.2%)	42 (33.6%)	35 (28%)	1 (0.8%)	23 (18.4%)	125 (100%)
15	Simulation tools are the best choice for teaching my subject area	23 (18.4%)	28 (22.4%)	61 (48.8%)	1 (0.8%)	12 (9.6%)	125 (100%)
16	My institute supports the integration of simulation	22 (17.6%)	43 (34.4%)	45 (36%)	2 (1.6%)	13 (10.4%)	125 (100%)
17	I have easy access to the facilities needed to assist me in the integration of simulation in my teaching	13 (10.4%)	33 (26.4%)	50 (40%)	5 (4%)	24 (19.2%)	125 (100%)
18	I need formal training to integrate simulation into the curriculum	29 (23.2%)	57 (45.6%)	17 (13.6%)	5 (4%)	17 (13.6%)	125 (100%)

SBE: Simulation-based Education

### First dimension: faculty members’ perceptions toward simulation-based education

Table 6 shows the arithmetic means and standard deviations of the sample responses sorted in descending order for each item of the first dimension. The general perception toward SBE was positive, with a mean score of 3.86 ± 0.74.

**Table 6:** Means and standard deviations of the sample responses sorted in descending for each Item of the first dimension

No.	Item	Mean	SD	Perception level	Order
4	Simulation-based teaching can improve learning outcomes.	4.07	0.96	Positive	1
3	Simulation is an effective assessment tool to evaluate students’ learning.	3.97	0.88	Positive	2
2	Students show more interest when I use simulation tools.	3.94	0.87	Positive	3
7	Simulation-based education improves patient safety.	3.86	0.90	Positive	4
1	I enjoy my teaching more when I use simulation.	3.80	0.90	Positive	5
6	Standardized patient is the best tool for teaching communication skills.	3.75	0.91	Positive	6
5	There is more freedom to learn in a simulated environment than learning in real wards or clinics.	3.61	0.99	Positive	7
**General perceptions level**		**3.86**	**0.74**	**Positive**	

### Second dimension: determine the anxiety of faculty members toward simulation-based education

Table 7 shows the arithmetic means and standard deviations of the sample responses sorted in descending order for each item of the second dimension. The general anxiety toward SBE was high, with a mean score of 3.42 ± 0.75.

**Table 7:** Means and standard deviations of the sample responses sorted in descending for each Item of the second dimension

No.	Item	Mean	SD	Anxiety level	Order
8	I need extra support to function effectively in simulation-based teaching.	3.84	0.87	High	1
12	Interaction with standardized patients makes students communicate in an artificial manner with real patients.	3.58	0.94	High	2
10	It takes more time to plan teaching with simulation tools rather than with real patients in student learning.	3.52	0.94	High	3
9	I face problems in managing students in simulated teaching.	3.18	0.83	Moderate	4
11	I avoid the integration of simulation in my courses.	2.99	1.06	Moderate	5
**General anxiety level**		**3.42**	**0.75**	**High**	

### Third dimension: faculty members’ perception toward the integration of simulation in education

Table 8 shows the arithmetic means and standard deviations of the sample responses sorted in descending order for each item of the third dimension. The general perception toward the integration of simulation was positive, with a mean score of 3.54 ± 0.79.

**Table 8:** Means and standard deviations of the sample responses sorted in descending for each Item of the third dimension

No.	Item	Mean	SD	Perception level	Order
13	Simulation should be a part of the medical curriculum, not a stand-alone activity.	3.78	1.02	Positive	1
18	I need formal training to integrate simulation into the curriculum.	3.70	1.09	Positive	2
16	My institute supports the integration of simulation.	3.56	0.95	Positive	3
14	Simulation-based activities should be introduced in the undergraduate curriculum from year 1.	3.52	1.03	Positive	4
15	Simulation tools are the best choice for teaching my subject area.	3.48	0.93	Positive	5
17	I have easy access to the facilities needed to assist me in the integration of simulation in my teaching.	3.20	1.00	Moderate	6
**General perceptions level**		**3.54**	**0.79**	**Positive**	

### Fourth dimension: faculty members’ perceived barriers towards the integration of simulation in education

Faculty members answered an open-ended question regarding perceived barriers they face towards SBE. The most frequent answers were the lack of time, training, facilities, resources, access, simulation labs, or simulation classrooms. The less frequent answers were the lack of cooperation, quick technical support, and motivation.

The answers only mentioned once were: the lack of training on implementation of the tool and the criteria for assessing, the lack of experience as they did not use the simulation at all, the difficulty in applying simulation/challenging to apply, the simulation was not integrated in the curriculum, and cultural barriers. One of the faculty mentioned that ‘there is always room for error, no matter how accurate the learning simulation is, there is always some scope for error and doubt when it comes to the re-creation of real-life scenarios, and the biggest drawback of using simulation is maintenance and updates can be costly. Besides these barriers, lack of time, labs, and enough training, particularly during COVID-19, were also mentioned.

Barriers related to the institution/university itself were reported, such as space, university environment, funding, lack of tools/materials, poor facilities, and support. The too busy schedule of the simulation center, insufficiently equipped laboratory, and the number of students per faculty; it is hard for one faculty to manage the students in simulation education, and lack of cooperation of leaders and other faculty members as it requires a lot of work early on and possibly a committee on its own to establish the rubrics for each subject and make the necessary refinement as needed. In addition, some community health departments are not using simulation as mentioned by the faculty, ‘it is unavailable in the department to be applied or used for the students.

Some of the faculties answered that no barriers were observed, but simulated education is not an original component of the curriculum and is only used as a backup.

## Discussion

Presented herein is the general perception and attitude of 125 faculty members about SBE application in the Colleges of Applied Medical Sciences in the Kingdom of Saudi Arabia. The general perception toward SBE was positive. The main findings of this study are that faculty members in the CAMS perceive SBE as an enjoyable way to teach, it increases students` interest and is considered an effective assessment tool to evaluate students` learning, improving learning outcomes, gives more freedom compared with actual wards and clinics, the best way for teaching communication skills, and improving patient safety. With a mean score of 3.86 ± 0.90. With this regard, the perception of faculty members toward SBE in this study is similar to students` perception in health colleges reported in other studies [1, 33, 34]. The students responded positively and believed that the simulations helped them better understand concepts, were a valuable learning experience, helped to stimulate critical thinking, and were realistic. The students also found that the knowledge gained from simulations can be transferred to real-life and clinical practice and that the simulations must be included in the undergraduate curriculum [33]. Many studies have demonstrated the importance of simulation as a teaching tool for healthy college students [33–35, 37]. The literature highlighted that students perceive SBE promotes learning outcomes [31, 32].

However, our results demonstrate that most CAMS faculty members have high levels of anxiety toward SBE. Faculty members agree and strongly agree with the statement that they need extra support to function effectively in simulation-based teaching and need more time to plan teaching with simulation tools compared with real patients. Unlike another study done in Saudi Arabia to evaluate the perception of medical teachers, their findings demonstrated that medical teachers have low levels of anxiety toward SBE [26]. The study compared the results for medical teachers in basic/clinical sciences, and they found that (59%/57.9%) disagree and strongly disagree with the statement that they avoid integrating simulation in their teaching [26]. Moreover, (42.3%/40.5%) of basic/clinical sciences medical teachers strongly agree that interaction with standardized patients will make the students interact artificially with actual patients.

Additionally, the results highlight that faculty members` perception was positive toward the integration of simulation in education. Faculty members agree and strongly agree with the statement that simulation should be a part of the medical curriculum and not a stand-alone activity, simulation-based activities should be introduced in the undergraduate curriculum from year 1, and simulation tools are the best choice for teaching their subject area. It has been demonstrated that the early introduction of SBE for basic clinical skills may simplify the integration of clinical and basic science knowledge, which may also increase student motivation and self-esteem [36, 38, 39].

Our findings also show that there was a statistically significant difference between the responses of the faculty members based on the training they received in simulation based on age, education level, and occupation. The rate of participants who received formal training for simulation was higher in 40–59 years (62.5%), holding a Master’s degree (65%) and working as Associate Professors and Professors (50%). Compared with the study that was done in Saudi Arabia to assess medical teachers` perception, the demographic factors of age, gender, received training, and perception of SBE, its anxiety, and integration were not statistically different [26].

Our study also identifies faculty members’ perceived barriers toward the integration of SBE, and the most frequently mentioned barriers were time, training, facilities, resources, access, and simulation labs/simulation classrooms. Compared to the other studies, curriculum design was one of the major barriers to the integration of SBE [26]. Another study aimed to assess the perceived barriers in applying simulation classes and reported the lack of interest to participate, lack of timely and effective feedback, and the high number of students in each class as perceived barriers to SBE [34].

This study has some strengths. First, this is a multicenter study; the population of participants was drawn from multiple institutions/universities in Saudi Arabia with different histories of curricular SBE. Thus, the findings can represent and generalize the perception of other institutions. Second, external validity and lower systematic bias compared to a single institution dataset. Third, to the best of our knowledge, this is the first study in Saudi Arabia that assessed faculty perception towards SBE in the community health sciences departments. Fourth, this study includes diverse faculty with various levels of experience and educational levels. Fifth, the second objective was to identify faculty members’ perceived barriers toward the integration of SBE, so this study offers the chance to identify the barriers and offer some solutions.

This study also has its limitations. Since data collection depended on a self-administered questionnaire, low response rates were the biggest challenges we faced. Other limitations related to self-administered questionnaires include misunderstanding, over/underestimation, and low monitoring ability. There is a possibility of selection and response bias even with anonymity since the faculty doesn’t have the same access or engagement with social media. The results can’t be generalized and may not be representative of the entire population of faculty members in Saudi Arabia to other programs because of the study design (cross-sectional). The interview was the best choice to take the expert opinion, but due to the lack of time and resources, we chose social media platforms and university emails to take the responses. As well, the sampling method we chose (snowball sampling) limited the ability to calculate the response rate. However, further observational and experimental studies are needed to assess the effect of SBE on learning and healthcare outcomes among students and healthcare practitioners.

## Conclusion

The study’s key results demonstrate the positive perception and attitude of medical teachers towards the SBE to be used for clinical nutrition undergraduate practical courses. However, the results show high levels of anxiety among faculty members toward SBE. For the effective use of simulation in practical courses, the study identifies the need for training, support, and evaluation of faculty members in SBE. Our findings highlight the barriers that CAMS administrations must address to support the integration of SBE in undergraduate practical courses, develop a faculty training program, and assess its impact. Further research is needed to be done in Saudi Arabia to demonstrate the SBE outcomes in the level of education and healthcare practice.

## Data Availability

The datasets used and analyzed during the current study are available from the corresponding author upon reasonable request.

## Abbreviations

CAMS: Colleges of Applied Medical Sciences
IRB: Institutional Review Board
OSCE: Objective Structured Clinical Examination
SBE: Simulation-based Education

## References

[1] AlBalawi I, Alqahtani JS, Al Ghamdi SS, Aldhahir AM, Alnasser M, Alqahtani AS et al. Health Sciences Students’ Attitude, Perception, and Experience of Using Educational Simulation in Saudi Arabia: A Cross-Sectional Study. Nurs Reports [Internet]. 2022 Sep 1 [cited 2023 Feb 11];12(3):620. Available from: /pmc/articles/PMC9501630/. 10.3390/nursrep12030061 PMC9501630 36135980

[2] Gaba DM. The future vision of simulation in health care. Qual Saf Health Care [Internet]. 2004 Oct 1 [cited 2023 Feb 4];13 Suppl 1(Suppl 1):i2–10. Available from: https://pubmed.ncbi.nlm.nih.gov/15465951/. 10.1136/qshc.2004.009878 PMC1765792 15465951

[3] Jeffries PR, Rodgers B, Adamson K. NLN Jeffries Simulation Theory: Brief Narrative Description. Nurs Educ Perspect [Internet]. 2015 [cited 2023 Feb 11];36(5):292–3. Available from: https://pubmed.ncbi.nlm.nih.gov/26521496/. 10.5480/1536-5026-36.5.292 26521496

[4] Aebersold M. The History of Simulation and Its Impact on the Future. AACN Adv Crit Care [Internet]. 2016 [cited 2023 Feb 11];27(1):56–61. Available from: https://pubmed.ncbi.nlm.nih.gov/26909454/. 10.4037/aacnacc2016436 26909454

[5] Aebersold M. Simulation-based learning: No longer a novelty in undergraduate education. Online J Issues Nurs [Internet]. 2018 May 1 [cited 2023 Feb 11];23(2):1–1. Available from: https://www.researchgate.net/publication/328628589_Simulation-Based_Learning_No_Longer_a_Novelty_in_Undergraduate_Education.

[6] Nestel D, Scerbo MW, Kardong-Edgren SE, A Contemporary History of Healthcare Simulation Research. Healthc Simul Res [Internet]. 2019 [cited 2022 Dec 14];9–14. Available from: https://link.springer.com/chapter/10.1007/978-3-030-26837-4_2.

[7] Rosen KR (2008). The history of medical simulation. J Crit Care.

[8] Adams CH, Fitz PA (1979). Simulation exercises for interview training in dietetics: a module on listening skills. J Am Diet Assoc.

[9] Caliendo MA, Pulaski FR. Teaching the health care team concept to dietetic and nursing students: Simulated team conferences. J Am Diet Assoc. 1979;74(5):571–3. 35560

[10] Sutnick MR, Carroll JG (1981). Using patient simulators to teach clinical interviewing skills. J Am Diet Assoc.

[11] Russell ML, Caggiula AW, Gloninger MF. Evaluation of clinical skills for nutrition counseling. J Am Coll Nutr [Internet]. 1985 Jan 1 [cited 2023 Jan 21];4(5):521–9. Available from: https://pubmed.ncbi.nlm.nih.gov/4056236/. 10.1080/07315724.1985.10720094 4056236

[12] Sayers SL (1986). Creating images of the future: a simulation game for dietetics students. J Am Diet Assoc.

[13] Stanek KL, Fox HM. A nutrition education workshop for managers of nutrition centers for the elderly utilizing videotaped simulations for assessment. J Nutr Elder [Internet]. 1989 Jul 31 [cited 2023 Jan 21];8(3–4):101–6. Available from: https://pubmed.ncbi.nlm.nih.gov/2769575/. 10.1300/J052v08n03_08 2769575

[14] Hampl JS, Herbold NH, Schneider MA, Sheeley AE. Using standardized patients to train and evaluate dietetics students. J Am Diet Assoc [Internet]. 1999 [cited 2022 Dec 25];99(9):1094–7. Available from: https://pubmed.ncbi.nlm.nih.gov/10491679/. 10.1016/S0002-8223(99)00261-8 10491679

[15] Boonmak P, Suraseranivongse S, Pattaravit N, Boonmak S, Jirativanont T, Lertbunnaphong T et al. Simulation-based medical education in Thailand: a cross-sectional online national survey. BMC Med Educ [Internet]. 2022 Dec 1 [cited 2022 Dec 26];22(1):1–9. Available from: https://bmcmededuc.biomedcentral.com/articles/10.1186/s12909-022-03369-9. 10.1186/s12909-022-03369-9 PMC9019967 35443707

[16] O’Shea MC, Palermo C, Rogers GD, Williams LT (2020). Simulation-based Learning experiences in Dietetics Programs: a systematic review. J Nutr Educ Behav.

[17] Gaba A, Costa SA, Schnoll R, Dorfman ME, Cordova S, Jakuboski S et al. Development and Evaluation of an Online Simulated Hospital Unit for Nutrition Assessment Training. Top Clin Nutr [Internet]. 2023 Apr 1 [cited 2023 May 21];38(2):133–43. Available from: https://journals.lww.com/topicsinclinicalnutrition/Fulltext/2023/04000/Development_and_Evaluation_of_an_Online_Simulated.6.aspx.

[18] Espinoza V, Marileo L, Viscardi S, Espinoza V, Marileo L, Viscardi S. Clinical simulation with dramatization, a teaching-learning strategy for undergraduate students of nutrition and dietetics. Arch Latinoam Nutr [Internet]. 2022 Jun 1 [cited 2023 Feb 11];72(2):93–9. Available from: http://ve.scielo.org/scielo.php?script=sci_arttext&pid=S0004-06222022000200093&lng=es&nrm=iso&tlng=en

[19] Moullet C, Depeyre J, Kehl C. [Diabetes simulation learning method for nutrition and dietetics students]. Rev Med Suisse [Internet]. 2021 Dec 1 [cited 2023 Feb 12];17(763):2209–12. Available from: https://europepmc.org/article/med/34910409. 34910409

[20] O’brien L, Collins K, Webb R, Davies I, Doran D, Amirabdollahian F. The Effects of Pre-Game Carbohydrate Intake on Running Performance and Substrate Utilisation during Simulated Gaelic Football Match Play. Nutr 2021, Vol 13, Page 1392 [Internet]. 2021 Apr 21 [cited 2023 Feb 12];13(5):1392. Available from: https://www.mdpi.com/2072-6643/13/5/1392/htm. 10.3390/nu13051392 PMC8142997 33919043

[21] Alkhaldy AA, Mosli RH. High-Fidelity Patient Simulation Increases Saudi Dietetics Students’ Self-efficacy in Applying the Nutrition Care Process. Top Clin Nutr [Internet]. 2020 Apr 1 [cited 2023 Jun 5];35(2):93–103. Available from: https://journals.lww.com/topicsinclinicalnutrition/Fulltext/2020/04000/High_Fidelity_Patient_Simulation_Increases_Saudi.2.aspx.

[22] BUCHHOLZ AC, Hendrickson M, Giroux I, CORREA JA, Hanning R, Eisenbraun C et al. Simulation in Learning and Using the Nutrition Care Process/Terminology: Experiences and Perceptions of Dietitians in Canada. Can J Diet Pract Res [Internet]. 2020 Sep 1 [cited 2023 Feb 12];81(3):150–3. Available from: https://pubmed.ncbi.nlm.nih.gov/32495644/. 10.3148/cjdpr-2020-010 32495644

[23] Tyler C, Alnaim L, Diekemper J, Hamilton-Reeves J, Goetz J, Sullivan DK et al. Simulations for Teaching and Evaluating Nutrition-Focused Physical Exam Skills. J Nutr Educ Behav [Internet]. 2020 Sep 1 [cited 2023 Feb 12];52(9):882–9. Available from: https://pubmed.ncbi.nlm.nih.gov/32446847/. 10.1016/j.jneb.2020.03.011 32446847

[24] Buchholz AC, Vanderleest K, MacMartin C, Prescod A, Wilson A. Patient Simulations Improve Dietetics Students’ and Interns’ Communication and Nutrition-Care Competence. J Nutr Educ Behav [Internet]. 2020 Apr 1 [cited 2023 Feb 12];52(4):377–84. Available from: https://pubmed.ncbi.nlm.nih.gov/31699616/. 10.1016/j.jneb.2019.09.022 31699616

[25] Steinhauser J, Janssen M, Hamm U. Who Buys Products with Nutrition and Health Claims? A Purchase Simulation with Eye Tracking on the Influence of Consumers’ Nutrition Knowledge and Health Motivation. Nutr 2019, Vol 11, Page 2199 [Internet]. 2019 Sep 12 [cited 2023 Feb 12];11(9):2199. Available from: https://www.mdpi.com/2072-6643/11/9/2199/htm. 10.3390/nu11092199 PMC6769812 31547369

[26] Ahmed S, Al-Mously N, Al-Senani F, Zafar M, Ahmed M. Medical teachers’ perception towards simulation-based medical education: A multicenter study in Saudi Arabia. Med Teach [Internet]. 2016 Mar 25 [cited 2023 Feb 2];38 Suppl 1:S37–44. Available from: https://pubmed.ncbi.nlm.nih.gov/26984032/. 10.3109/0142159X.2016.1142513 26984032

[27] Morey JC, Simon R, Jay GD, Wears RL, Salisbury M, Dukes KA et al. Error Reduction and Performance Improvement in the Emergency Department through Formal Teamwork Training: Evaluation Results of the MedTeams Project. Health Serv Res [Internet]. 2002 [cited 2023 Jan 22];37(6):1553. Available from: /pmc/articles/PMC1464040/. 10.1111/1475-6773.01104 PMC1464040 12546286

[28] Erdfelder E, FAul F, Buchner A, Lang AG. Statistical power analyses using G*Power 3.1: tests for correlation and regression analyses. Behav Res Methods [Internet]. 2009 [cited 2023 Jun 12];41(4):1149–60. Available from: https://pubmed.ncbi.nlm.nih.gov/19897823/. 10.3758/BRM.41.4.1149 19897823

[29] Ziv A, Root Wolpe P, Small SD, Glick S. Simulation-based medical education: an ethical imperative. Acad Med [Internet]. 2003 Aug 1 [cited 2023 Feb 4];78(8):783–8. Available from: https://pubmed.ncbi.nlm.nih.gov/12915366/. 10.1097/00001888-200308000-00006 12915366

[30] Issenberg SB, McGaghie WC, Petrusa ER, Gordon DL, Scalese RJ. Features and uses of high-fidelity medical simulations that lead to effective learning: a BEME systematic review. Med Teach [Internet]. 2005 Jan [cited 2023 Feb 4];27(1):10–28. Available from: https://pubmed.ncbi.nlm.nih.gov/16147767/. 10.1080/01421590500046924 16147767

[31] McGaghie WC, Issenberg SB, Petrusa ER, Scalese RJ. A critical review of simulation-based medical education research: 2003–2009. Med Educ [Internet]. 2010 Jan [cited 2023 Feb 4];44(1):50–63. Available from: https://pubmed.ncbi.nlm.nih.gov/20078756/. 10.1111/j.1365-2923.2009.03547.x 20078756

[32] Taber KS, The Use of Cronbach’s Alpha When Developing and Reporting Research Instruments in Science Education. Res Sci Educ [Internet]. 2018 Dec 1 [cited 2023 Jun 9];48(6):1273–96. Available from: https://link.springer.com/article/10.1007/s11165-016-9602-2.

[33] Howard VM, Englert N, Kameg K, Perozzi K (2011). Integration of Simulation across the undergraduate curriculum: Student and Faculty perspectives. Clin Simul Nurs.

[34] Nuzhat A, Salem RO, Al Shehri FN, Al Hamdan N. Role and challenges of simulation in undergraduate curriculum. Med Teach [Internet]. 2014 [cited 2023 Jun 9];36 Suppl 1(SUPPL.1). Available from: https://pubmed.ncbi.nlm.nih.gov/24617788/. 10.3109/0142159X.2014.886017 24617788

[35] Issenberg SB. The scope of simulation-based healthcare education. Simul Healthc [Internet]. 2006 [cited 2023 Jun 9];1(4):203–8. Available from: https://journals.lww.com/simulationinhealthcare/Fulltext/2006/00140/The_Scope_of_Simulation_based_Healthcare_Education.1.aspx. 10.1097/01.SIH.0000246607.36504.5a 19088590

[36] McGaghie WC. Research opportunities in simulation-based medical education using deliberate practice. Acad Emerg Med [Internet]. 2008 Nov [cited 2023 Jun 9];15(11):995–1001. Available from: https://pubmed.ncbi.nlm.nih.gov/18811635/. 10.1111/j.1553-2712.2008.00246.x 18811635

[37] AlMously N, Baalash A, Salem R, Mukaddam S. The proper timing to introduce simulation-based education in internal medicine clerkship. J Contemp Med Educ [Internet]. 2014 [cited 2023 Jun 9];2(3):180. Available from: https://www.researchgate.net/publication/287537558_The_proper_timing_to_introduce_simulation-based_education_in_internal_medicine_clerkship.

[38] Valler-Jones T, Meechan R, Jones H. Simulated practice–a panacea for health education? Br J Nurs [Internet]. 2011 May 26 [cited 2023 Jun 9];20(10):628–31. Available from: https://pubmed.ncbi.nlm.nih.gov/21646995/. 10.12968/bjon.2011.20.10.628 21646995

[39] K AD, Kadir SY, Viswanathan N. Early introduction of clinical skills in the pre-clinical phase of an integrated medical curriculum. J Contemp Med Educ [Internet]. 2013 Jul 10 [cited 2023 Jun 9];1(3):142–4. Available from: https://www.jcmedu.org/abstract/early-introduction-of-clinical-skills-in-the-preclinical-phase-of-an-integrated-medical-curriculum-48446.html.

